# A51 DEFINING THE ROLE OF THE SPDEF TRANSCRIPTION FACTOR IN INTESTINAL INFLAMMATION

**DOI:** 10.1093/jcag/gwae059.051

**Published:** 2025-02-10

**Authors:** A Jamal, H Wang, J Grondin, T Seto, A Kamal, W I Khan

**Affiliations:** Pathology and Molecular Medicine, McMaster University, Hamilton, ON, Canada; Pathology and Molecular Medicine, McMaster University, Hamilton, ON, Canada; Pathology and Molecular Medicine, McMaster University, Hamilton, ON, Canada; Pathology and Molecular Medicine, McMaster University, Hamilton, ON, Canada; Pathology and Molecular Medicine, McMaster University, Hamilton, ON, Canada; Pathology and Molecular Medicine, McMaster University, Hamilton, ON, Canada

## Abstract

**Background:**

The intestinal mucus layer prevents commensal microbes and luminal pathogens from interacting with and invading into the underlying epithelium. Goblet cells are predominantly responsible for maintaining this mucus layer through the production and secretion of mucins. Alterations in goblet cell responses are observed in various gastrointestinal disorders including inflammatory bowel disease (IBD). The differentiation and maturation of goblet cells is controlled by several genes. Recently it was shown that the SAM pointed domain ETS factor (SPDEF), an ETS family transcription factor plays a key role in terminal differentiation and maturation of goblet cells. The role of SPDEF in goblet cell responses in intestinal inflammation remains to be determined. Understanding its role in regulating goblet cell responses and mucins production will provide new insight into the mechanisms of dysregulated mucus barrier in intestinal inflammation.

**Aims:**

This study aimed to investigate the severity of a commonly employed chemical model of colitis, dextran sodium sulphate (DSS), in SPDEF knockout (KO) mice compared to wild-type (WT) littermate controls. It was hypothesized that the SPDEF KO mice will exhibit a heightened severity of DSS-induced colitis than the WT mice.

**Methods:**

SPDEF KO and WT mice were treated with DSS in the drinking water for seven consecutive days then euthanized. Disease activity index (DAI), comprising of body weight, stool consistency, and fecal bleeding was assessed over the course of DSS administration. The severity of intestinal inflammation was further assessed by checking macroscopic scores, histological damage scores, myeloperoxidase (MPO) assay, and various pro-inflammatory cytokines in the colonic tissues by ELISA.

**Results:**

SPDEF KO mice exhibited a heightened sensitivity to DSS-induced colitis compared to WT controls. The SPDEF KO mice show a much more dramatic decline in body weight and a substantial increase in DAI, most prominently during the later stages of DSS administration. Additionally, SPDEF KO mice exhibited a greater degree of colonic, fecal, and rectal bleeding than the WT mice, reflected by a higher macroscopic score. Levels of MPO and certain pro-inflammatory cytokines were significantly elevated in the colon tissues of SPDEF KO as compared to WT mice. Histologically, SPDEF KO also displayed decreased goblet cell counts compared to WT mice.

**Conclusions:**

These findings suggest that disruption of the SPDEF transcription factor aggravates DSS-colitis. Defining the role of SPDEF in intestinal inflammation provides a deeper understanding of the mechanism of dysregulated mucus barrier in colitis which may ultimately lead to identifying potential new targets to restore barrier integrity in colitis.

**Funding Agencies:**

NRC

